# Characterization of malaria transmission by vector populations for improved interventions during the dry season in the Kpone-on-Sea area of coastal Ghana

**DOI:** 10.1186/1756-3305-5-212

**Published:** 2012-09-26

**Authors:** David P Tchouassi, Isabella A Quakyi, Ebenezer A Addison, Kwabena M Bosompem, Michael D Wilson, Maxwell A Appawu, Charles A Brown, Daniel A Boakye

**Affiliations:** 1 School of Public Health, University of Ghana, Legon, Ghana; 2 Noguchi Memorial Institute for Medical Research, University of Ghana, Legon, Ghana

**Keywords:** *Anopheles gambiae* M molecular form, *Plasmodium falciparum*, Malaria transmission, Biting pattern, Parity rate, EIR, Ghana

## Abstract

**Background:**

Malaria is a major public health problem in Ghana. We present a site-specific entomological study of malaria vectors and transmission indices as part of an effort to develop a site for the testing of improved control strategies including possible vaccine trials.

**Methods:**

Pyrethrum spray catches (PSC), and indoor and outdoor human landing collections of adult female anopheline mosquitoes were carried out over a six-month period (November 2005 - April 2006) at Kpone-on-Sea, a fishing village in southern Ghana. These were morphologically identified to species level and sibling species of the *Anopheles gambiae* complex further characterized by the polymerase chain reaction (PCR). Enzyme-linked immunosorbent assay was used to detect *Plasmodium falciparum* mosquito infectivity and host blood meal sources. Parity rate was examined based on dilatation of ovarian tracheoles following dissection.

**Results:**

Of the 1233 *Anopheles* mosquitoes collected, *An. gambiae* s.l. was predominant (99.5%), followed by *An. funestus* (0.4%) and *An. pharoensis* (0.1%). All *An. gambiae* s.l. examined (480) were identified as *An. gambiae* s.s. with a majority of M molecular form (98.2%) and only 1.8% S form with no record of M/S hybrid. A significantly higher proportion of anophelines were observed outdoors relative to indoors (*χ*^2^ = 159.34, df = 1, p < 0.0000). Only *An. gambiae* M molecular form contributed to transmission with a high degree of anthropophily, parity rate and an estimated entomological inoculation rate (EIR) of 62.1 infective bites/person/year. The Majority of the infective bites occurred outdoors after 09.00 pm reaching peaks between 12.00-01.00 am and 03.00-04.00 am.

**Conclusion:**

*Anopheles gambiae* M molecular form is responsible for maintaining the status quo of malaria in the surveyed site during the study period. The findings provide a baseline for evidence-based planning and implementation of improved malaria interventions. The plasticity observed in biting patterns especially the combined outdoor and early biting behavior of the vector may undermine the success of insecticide-based strategies using insecticide treated nets (ITN) and indoor residual spray (IRS). As such, novel or improved vector interventions should be informed by the local malaria epidemiology data as it relates to vector behavior.

## Background

The burden of malaria remains high in many African countries, despite increasing effort to control the disease due to a persistent high level of transmission [1,2]. Determining the intensity of transmission by mosquito populations is a key component of epidemiologic studies of malaria. This is usually estimated using the entomological inoculation rate (EIR), an index which provides the most direct measure of the risk of human exposure to the bites of infective anopheline vectors [3]. The EIR is also highly valuable for monitoring the suitability of vector control operations [4]. The risk of human exposure to infectious bites of vectors in Africa is however, not uniform [5]. Indeed, the transmission pattern may even vary greatly from region to region and even from village to village in the same district [6-8].

In most malarious regions of the world, there is little baseline information on vector populations and variation in the intensity of malaria transmission. Consequently, currently used vector control methods via indoor residual sprays (IRS) and Insecticide treated bed nets (ITNs) are applied without regard to the local epidemiology of the disease, especially the relationship to vector behavior and intensity of transmission. Optimum effectiveness of these control strategies presumably depends on vectors biting at hours when most people are in bed [9]. However, behavioral heterogeneity of *Anopheles* species in ecologically different localities is widespread [10] and could dictate the success of these strategies. For example, The Garki malaria control project in Nigeria in the 1970s failed largely because of failure to recognize persistent malaria transmission by exophilic outdoor-resting mosquitoes, despite widespread indoor residual insecticide spraying [11-13].

Therefore, the implementation of effective vector control strategies requires information on the main vectors, their population structure, distribution and efficiency in malaria transmission and variation even within local scales [1]. Moreover, entomological parameters to identify the main vectors for selecting suitable vector control options [9,14,15] are required in several communities in Africa, including Ghana, where malaria remains endemic in several communities [16,17].

Efforts are being made to achieve malaria elimination and eradication worldwide [18,19]. The strategies being adopted include improved vector control, chemotherapy and possible vaccination. Kpone-on-Sea, a coastal fishing village in southern Ghana, is being developed as a possible site for testing various malaria control strategies with the hope to improve case management, control and prevention of the disease. To achieve effectiveness in these strategies, all aspects of malaria epidemiology need to be well understood including a better understanding of the vector transmission indices. Thus, this paper describes an entomological study on the vectors of malaria and their relative contributions to *Plasmodium falciparum* transmission at Kpone-on-Sea. The study was conducted over a period of six months (November 2005 - April 2006) during the dry season especially where vectors are likely to be confronted with highly variable and challenging climatic conditions with likely change on the disease transmission patterns.

## Methods

### Study area: kpone-on-Sea

Kpone-on-Sea is a fishing village situated at 5°69’N, 0°06’E within the coastal savanna belt of West Africa. It is bordered on the East by Prampram, on the West by Tema, on the South by the Gulf of Guinea (Atlantic Ocean), and on the North by shrub land, beyond which is the Ghana Industrial Free Zone. It is at an altitude of 50–100 m above sea level and has an equatorial climate. The village is located in the Tema Municipal Health Directorate, within the Greater Accra Region of Ghana. Temperatures range from 24.4°C-27.8°C with a mean of 26.1°C. Mean annual rainfall averages between 1133 and 3606 mm with an average relative humidity index ranging from 78% to 85%. The land formation and the drainage patterns of the four sectors of the village are such that all water from the village drains into a stream that lies on the outskirts of the village. There is also a lagoon on the outskirts of the village. A recent study in the village showed a low prevalence of malaria (11%) with a peak parasite rate of 21% in children aged 1–5 years. *Plasmodium falciparum* was the major parasite detected in all positive blood slide examinations [20]. Most of the houses are constructed of cement and corrugated iron roofing. The majority of the residents (80%) are of the Ga and Ga-Adangbe ethnic groups. Most of the inhabitants are fishermen and a sizeable proportion involved in vegetable farming.

### Field sampling of mosquitoes, morphological identification and parity determination

Adult mosquito surveys were carried out in the village using indoor and outdoor human landing catches (HLC) from 18:00–06:00 and indoor pyrethrum spray catches (PSC) from 06:00–08:00 (WHO, 1975). Both indoor and outdoor HLCs were conducted four nights per month (once weekly), for 6 months during the dry season from November 2005 to April 2006, in two randomly selected houses, at least 40 m apart by two teams of four collectors each with a supervisor. The collectors worked in pairs with a personnel change at midnight, one pair working from 6:00 pm to midnight and the next from midnight until 6:00 am. At each house, a collector was posted indoors and another outdoors with a flashlight and a mouth aspirator. Collection teams were rotated among blocks each month to limit temporal and/or collector bias. Each month, before continuing surveys of non-sampled households, an attempt was made to inspect premises that were previously closed or where access had been refused. Access to these areas was attempted at least three times. Different pairs of houses were visited every month. Collected mosquitoes were sorted out and the female anophelines morphologically identified using taxonomic keys [21,22]. The ovaries of all fresh unfed specimens were dissected and examined for parity determination [23]. The carcasses of each dissected mosquito were preserved individually on cotton wool over a desiccant (silica gel) in labeled Eppendorf tubes and stored at −20°C for immunological and molecular biological analyses.

### PCR identification of the *Anopheles gambiae* complex

Genomic DNA of *An. gambiae* s.l. mosquitoes was extracted by homogenizing a mosquito leg in 50 μl of sterile double distilled water in 1.5 ml Eppendorf tube using sterile plastic pestles. The homogenates were then boiled for 10 minutes, allowed to cool and kept at −20°C until required. The PCR method of Scott *et al*. [24] was used for the identification of the sibling species of the *An. gambiae* complex. A fraction of the *Anopheles gambiae* s.s. specimens were further analyzed to determine the M and S molecular forms as described by Favia *et al*. [25]. The digests were visualized in ethidium bromide stained 2% agarose gels.

### Determination of *P. falciparum* sporozoite infections and human blood index

The head and thorax of each mosquito were separated from the rest of the body, homogenized in blocking buffer (0.5% Casein, 0.1 N NaOH, 1x PBS) and a portion of the homogenate assayed by ELISA for the presence of circumsporozoite antigens (CSA) of *P. falciparum* as described by Wirtz *et al*. [26]. Positive controls (Kikergaard & Perry Laboratories, USA) and negative controls (uninfected laboratory reared mosquitoes) were assayed simultaneously. A specimen was considered positive if a visual green colour was detected with an optical density (OD) value (at 405 nm) of at least the mean of the negative controls plus two standard deviations.

Blood-fed *Anopheles* species from house-resting collections (i.e., PSC) were tested for the source of the blood meals using alkaline phosphatase–conjugated immunoglobulin Gs (IgGs) of human, goat, and bovine (Sigma Co., St. Louis, MO, USA). The ELISA results were read visually according to the protocol of Beier *et al.*[27]. The human blood index (HBI) was calculated as the proportion of blood-fed mosquitoes that had fed on humans out of the total tested.

### Data analyses

Entomological parameters considered were: 1) Man-biting rate, calculated as the number of bites received per person per night of collection using the formula by Lines *et al.*[28]; 2) Infection rate, measured as the proportion of mosquitoes found to contain circumsporozoite antigen (CSA) by ELISA; 3) Parity rate, measured as the ratio of parous mosquitoes to the total of parous and nulliparous mosquitoes dissected; 4) Entomological inoculation rate (EIR), derived as the product of the man biting rate and circumsporozoite antigen rate as determined by ELISA; 5) The human blood index (HBI), which is the proportion of mosquitoes found to contain human IgG by ELISA. Differences in the abundance of *Anopheles* mosquitoes (indoor and outdoor) were analyzed using the chi-square goodness-of-fit test at P = 0.05 level of significance using R statistical software [29].

### Ethical considerations

Informed consent from all the participants and ethical approval from the Noguchi Memorial Institute for Medical Research Institutional Review Board (NMIMR-IRB) was obtained. A sensitization rally was organized with the population during which the purpose of the study was clearly explained. Free informed consent of volunteers (to participate in mosquito collections) and heads of families was requested through individual discussions and group meetings, prior to the enrolment of their house in the study. Presumptive malaria treatment was given throughout the course of the study to volunteers as recommended by the National Malaria Control Programme.

## Results

### Anopheline species abundance

A total of 1,233 *Anopheles* mosquitoes were collected during the study period. Of these, *An. gambiae* s.l. constituted 99.5%, followed by *An. funestus* (0.4%) and of *An. pharoensis* (0.1%). Table1 shows the species composition by the different collection methods.

**Table 1:** **Total captures of *Anopheles *mosquitoes by collection methods at Kpone-on-Sea **

**Collection method**	***Anopheles *species**			**Total**
	***An. gambiae *s.l.**	***An. funestus***	***An. pharoensis***	
HLC (Indoor)	383 (31.68)	4 (80)	0	387
HLC (Outdoor)	826 (68.32)	1 (20)	1 (100)	828
PSC (Indoor)	18 (100)	0	0	18
Total	1227	5	1	1233

HLC, human landing catches; PSC, pyrethrum spray catches; Numbers in parentheses are percentages.

### Species and molecular forms of *Anopheles gambiae* s.s

A total of 480 (inclusive of the 15 samples that were positive for CSA) out of 1,209 morphologically determined *An. gambiae* s.l. specimens were identified further by PCR as *An. gambiae* s.s. Molecular forms of approximately a tenth of the identified *An. gambiae* s.s. (inclusive of the 15 samples positive for CSA) were further determined. The M-form constituted 98.2% while the S-form constituted 1.8% (n = 56). All the samples, which showed positive for CSA of *P. falciparum* where of the M-molecular form *An. gambiae* s.s.

**Figure 1:**
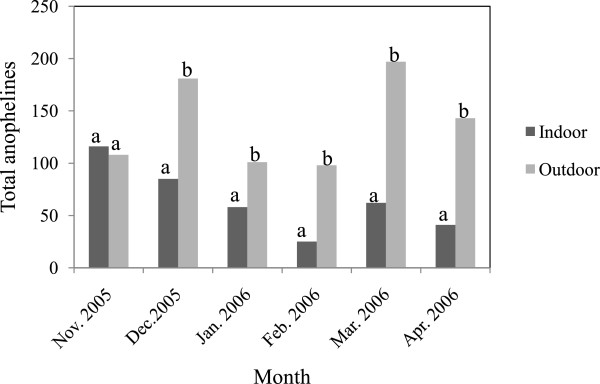
**Monthly captures and comparisons of total anophelines by Human Landing Catches during the dry season.** Bars followed by same letters are not significantly different at p = 0.05.

Figure1 shows the monthly indoor and outdoor species composition of the anopheline mosquitoes caught throughout the study period. Except in November 2005, a higher proportion of *Anopheles* mosquitoes were observed outdoors than indoors during all the months throughout the study period and the overall proportion of anopheline mosquitoes caught outdoors (68.15%) was significantly higher than those caught indoors (31.85%) (*χ*^2^ = 159.34, df = 1, p < 0.0000).

### Man-biting and parity rates

The overall (indoor and outdoor) mean biting rates of *An. gambiae* s.l. was 11.37 bites/human/night (b/m/n). Only five *An. funestus* (0.05 b/m/n) and one *An. pharoensis* (0.01b/m/n) were caught biting throughout the study period. The overall biting rate due to all three anopheline species for the entire period was 11.43 b/m/n (Table2). The overall mean parity rate calculated for *An. gambiae* s.l. was 79.9% (n = 1209). Of the five *An. funestus* sampled, four were parous, thereby recording a parous rate of 80%. The only *An. pharoensis* captured was nulliparous. The overall parity rate for the study period was 80.25% (n = 1215) (Table3).

**Table 2:** **Entomological indices estimated for *Anopheles *mosquitoes collected at Kpone-on-Sea**

***Anopheles *species**	**No. (%)**	**Entomological indices**			
		**MBR (b/m/n)**	** CSA (n)**	** HBI (n)**	**EIR**
*An. gambiae* s.l.	1209 (99.51)	11.37	0.015 (971)	67.67 (18)	0.17
*An. funestus*	5 (0.41)	0.03	0	0	0
*An. pharoensis*	1 (0.08)	0	0	0	0

MBR , man biting rate; b/m/n, bite/man/night; CSA, circumsporozoite antigen rate (%); HBI, human blood index (%); EIR, entomological inoculation rate; (n), number examined.

**Table 3:** Monthly parity rates of anopheline mosquitoes at Kpone-on-Sea

***Anopheles *species**	**Study period**					
	**November 2005**	**December 2005**	**January 2006**	**February 2006**	**March 2006**	**April 2006**
*An. gambiae* s.l.	91.07 (224)	87.97 (266)	78.06 (155)	62.60 (123)	72.37 (258)	78.69 (183)
*An. funestus*	0	0	75 (4)	0	100 (1)	0
*An. pharoensis*	0	0	0	0	0	0 (1)

Numbers in brackets indicate numbers examined.

### Sporozoite and entomological inoculation rates and biting patterns

Fifteen out of the 984 *An. gambiae* s.s. examined were infected, giving a CSA rate of 1.52%. None of the five *An. funestus* examined were positive. Only *An. gambiae* s.s. was found to be infective and of the molecular M-form, thus incriminating this species as the sole vector in the study site during this period. Fourteen out of the 15 infections occurred in the HLC samples, whilst one was in the PSC samples. Of the fourteen HLC infections, eleven occurred in outdoor samples and three in indoor samples. All the infective HLC mosquitoes were caught between 9.00 pm and 5.00 am. Hourly distribution of sporozoite-positive bites of *An. gambiae* s.s. in the study area revealed that most of the infective bites occurred after 21.00 hrs, reaching their peak between 12.00 - 01.00 am and 03.00 - 04.00 am. No infective bites were recorded between 18.00 and 20.00 hrs. Of the total mosquitoes found to be infective, eight were caught in November 2005, one in December 2005, one in January 2006, two in February 2006, two in March 2006 and one in April 2006.

The estimated mean daily *P. falciparum* EIR or the mean number of infective bites per man per night (ib/m/n) for *An. gambiae* s.s. was 0.17 (Table2). Thus within a year, inhabitants in this area would have received an average of 62.1 infective bites at Kpone- on-Sea, if no precautionary measures had been taken. There were also monthly fluctuations in the EIR with the period of greatest risk to humans from infective bites of *An. gambiae* s.l. recorded in November 2005 and February 2006.

**Figure 2:**
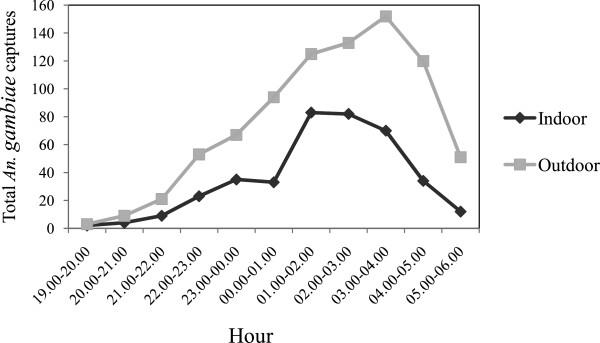
**Hourly biting patterns of *An. gambiae *s.s. by Human Landing Catches during the dry season**.

Biting by *An. gambiae* commenced early in the evening and continued till daybreak (06.00–07.00 am) (Figure2). Peak biting activity was observed between the hours of 01:00–02:00 and 02:00–03:00 both indoor and outdoor. Biting by *An. gambiae* s.s. was highest in December 2005 and coincided with the month in which the highest number of this species was caught probably as a result of slight showers that occurred prior to sampling during the month (Figure1).

### Human blood index (HBI)

*Culex* species formed the dominant species collected by the PSC accounting for 87.32% (n = 124) of the 142 mosquitoes collected (data not shown). Only a total of 18 blood-fed indoor resting adult *An. gambiae* s.s. caught were studied and the estimated HBI of this species was 66.67%. Out of these, 22.22% (4/18) were observed to have fed on goats while none had fed on cattle. The remaining 11.11% were observed to have neither fed on humans nor goats. There were no reagents to test for other possible host blood meal sources.

## Discussion

*Anopheles gambiae* s.s. was the dominant anopheline mosquito and the main sibling species of the *An. gambiae* complex in the surveyed area. This concurs with findings in coastal savanna areas of Ghana [30,31] and elsewhere in Africa [32]. *Anopheles melas* was absent amongst the human biting populations of the *An. gambiae* complex, although it is known to be a vector along the coast of West Africa [33-35]*.* This species therefore, appears not to play a role in malaria transmission in the coastal areas of Ghana. Other anophelines recorded but in reduced numbers included *An. funestus* and *An. pharoensis*, a finding which is similar to earlier work in the nearby coastal savanna area of Prampram [30].

The *Anopheles gambiae* s.s. M molecular form was dominant and the main malaria vector identified. The molecular M-form has been reported to have a higher vectorial capacity [36]. This fact is corroborated in our findings where a high circumsporozoite antigen rate was recorded and which was relatively higher compared to earlier studies in coastal savanna areas of Ghana [30,31]. The M-form is known to breed in permanent and semi-permanent water swamps in floodable river banks favorable to its development [37]. The low occurrence of the molecular S-form in our study during the dry season is also not surprising as this form is known to be well adapted to rainfall breeding sites [38,39]. Although these molecular forms are thought to represent incipient species [40,41] the factors underlining their co-existence in this area are still unclear, and deserve further investigation.

Peak indoor infective bites were also observed between the hours of 12.00 - 01.00 am when most of the inhabitants were in bed; hence suggesting that if inhabitants slept under impregnated bednets, human-vector contact and thus the risk of infective bites could be reduced. However, the majority of the infective bites occurred outdoors between the hours of 12.00-01.00 am and 03.00-04.00 am which coincides with peak biting densities of this vector. An interesting finding was the distribution of sporozoite-laden bites during the night, which indicated that malaria transmission in the study site occurred in the evenings after 09.00 pm till near daybreak (04.00 - 05.00 am). This has important epidemiologic implications as some of the inhabitants, involved in domestic and other activities are already out of bed (by 04.00 am) so therefore, increases their exposure to infective inoculations.

The high degree of outdoor behavior contrasts with a greater endophilic tendency displayed by this species in earlier studies in nearby coastal area of Ghana [30]. This observation is however, not surprising as heterogeneity in biting pattern is common place even within local scales. Equally, early evening biting activity by *An. gambiae* s.s. was evident, a pattern which contrasts with that reported by [30] where no biting occurred during the early hours of the morning. The basis for the combined high degree of outdoor biting and early biting populations remains unclear, but could be in response to prolonged use of insecticides indoors [9]. Such characteristics would tend to reduce the impact of control strategies directed towards the indoor biting fraction of the population.

This variation in feeding behavior within vector species may have a genetic basis [11], which raises the possibility that vector control measures using insecticides could select for genotypes which are least likely to encounter the intervention. The early and outdoor biting populations may represent behavioral shifts as a consequence of phenotypic plasticity or evolutionary change within vector populations which remains unclear [42]. Regardless of the mechanism, such behavioral plasticity limits contact between vectors and insecticides, thus diminishing the effectiveness of the interventions that use them [9,43]. This outdoor biting activity has been linked to persistent malaria transmission in the face of mounting control measures indoors [42]. Thus there have been calls for innovative measures to develop new tools to fight malaria transmission by exploiting the ecology of the vector through an integrated approach to complement current vector control strategies [19,42,44].

No infected *An. funestus* was recorded during the study period. Previous studies in other coastal areas of Ghana [30,31] have implicated this species as the second most important vector after *An. gambiae* s.l. Therefore, the absence of infection in *An. funestus* might be attributable to the low numbers caught biting; hence a longer period of survey in the rainy season is required to ascertain whether *An. funestus* is a significant vector at Kpone-on-Sea.

The observed parity rates for the anophelines were high. This indicates that older populations of mosquitoes tend to accumulate with time. This allows for increased feeding frequencies and thus, increased chances of the vectors becoming infected or even re-infected during subsequent feeding [45,46]. Our analysis showed that a higher proportion of the engorged *An. gambiae* had fed on humans. This finding confirms the anthropophilic tendencies displayed by this species throughout most of its distribution [47], making it the most efficient malaria vector in Africa. Host blood meal detection assay was, however, conducted on only the few number of anophelines sampled using PSC. Inefficiencies of collectors could introduce bias in the number of mosquitoes that have fed on humans using human landing catches. As such, only anopheline mosquitoes collected by PSC were assayed for blood meal analyses. Few anophelines were captured using this strategy although the reason for the low captures is unclear given adequate sampling regimes that were conducted. It has been noted however, that during the dry season, as a survival strategy, adults may hide in shelters such as rodent burrows, abandoned houses and wells, thereby minimizing the chances of their detection through pyrethrum spray collections [48].

Malaria transmission dynamics have been shown to vary greatly across Africa with inoculation rates varying from as low as 0.1 to over 1000 infective bites/person/year (ib/p/y) [7,49]. Generally in Africa, when the EIR <10, the area is considered to have unstable malaria and where EIR > 100, malaria is said to be stable [50]. Our results therefore suggest that malaria endemicity at Kpone-on-Sea remains variable, which depends on environmental and demographic conditions such as rainfall, vegetation cover, human population density and land use patterns. The observed annual inoculation rates in Kpone-on-Sea is, however, higher relative to other coastal areas of Ghana [30,31] probably due to a multiplier effect of the density and high biting rate of this species*.*

## Conclusion

The findings provide a baseline for evidence-based planning and implementation of malaria control activities targeting vectors. *An. gambiae* s.s. is responsible for maintaining the status quo of malaria in the study site during the study period. The plasticity observed in biting patterns, especially the combined outdoor and early biting behavior of the vector has important consequences for the success of the widely used insecticide-based strategies using ITN and IRS. New or improved interventions should be informed by the local malaria epidemiology as it relates to vector behavior.

## Competing interests

The authors declare that they have no competing interests.

## Authors’ contributions

DPT IAQ MDW DAB conceived and designed experiments. DPT conducted the experimental work. DPT analyzed the data. DPT IAQ EAA MDW MAA CAB DAB contributed to the manuscript. All authors approved the final version of the manuscript.
